# Comparative Study on Residents' Health-Promoting Lifestyle and Life Satisfaction in Wuhan Before and After the COVID-19 Pandemic

**DOI:** 10.3389/fpubh.2022.820499

**Published:** 2022-02-23

**Authors:** Da Ke, Wei Chen

**Affiliations:** College of Physical Education, Wuhan Sports University, Wuhan, China

**Keywords:** health-promoting lifestyle, life satisfaction, residents, Wuhan, COVID-19 pandemic

## Abstract

The Coronavirus Disease-19 (COVID-19) pandemic has dramatically affected residents' life. Whether the COVID-19 pandemic has significantly influenced the residents' health-promoting lifestyle, and life satisfaction is an urgent problem to be studied. Based on Health Belief Model (HBM), this paper explored and compared the responses of residents' health-promoting lifestyle and life satisfaction on the influence of the COVID-19 pandemic. Data were collected from a sample of 2,054 residents in Wuhan by questionnaire survey. The results show that the total score of health-promoting lifestyle after the COVID-19 pandemic has increased significantly compared with that before the COVID-19 pandemic, and the scores of all dimensions of health-promoting lifestyle have improved. Among them, the scores of exercises, self-actualization, and stress management are significantly higher than those before the COVID-19 pandemic. However, the score of residents' life satisfaction has shown a downward trend. There were also significant differences in life satisfaction on the demographic variables, such as gender, age, education level, marital status, and family average income. The findings are of great significance in promoting residents' health-promoting lifestyles and life satisfaction in the context of the extraordinary pandemic.

## Introduction

The importance of the health-promoting lifestyle in maintaining personal health has been highlighted (1–4). The concept of a health-promoting lifestyle has been initially put forward by Walker et al. (4), which is also known as healthy behavior. It refers to a multidimensional model of self-initiated actions and perceptions on health, which serve to preserve or enhance people's wellness, self-actualization, and fulfillment. Health-promoting lifestyle mainly contains six dimensions, such as self-actualization, health responsibility, exercise, nutrition, interpersonal support, and stress management (4).

To date, extensive studies have been shown that residents with a health-promoting lifestyle would follow with interest to their health status and disease prevention (1). There is a significant positive correlation between health-promoting lifestyle and residents' physical health, mental health, and social interactions (2). Physiological factors, psychological factors, and cognitive factors have a significant effect on health-promoting lifestyles, such as school education and environment (3), age, physical condition (5), family (6), marital status, education level, income (7), and individual psychology (5, 8, 9).

Moreover, previous studies have identified that people's health-promoting lifestyle would significantly affect the quality of life satisfaction (10). Life satisfaction, which is an essential part of subjective wellbeing, is defined as people's independent judgment and evaluation of their life happiness (11). An unreasonable health-promoting lifestyle would increase the probability of illness and reduce physical and psychological life satisfaction. Carrying out the appropriate intervention programs of health-promoting lifestyle could effectively improve physical, mental health, and life satisfaction (12–14). Individuals with different levels of life satisfaction would also have different health-promoting lifestyles (15). How to keep a healthy lifestyle and high life satisfaction has been a question of great interest.

Nowadays, the emergence of the Coronavirus Disease-2019 (COVID-19) pandemic, which has been defined as a public health emergency, has directly threatened people's health. Although the COVID-19 pandemic has been controlled to some extent, the effect of the COVID-19 pandemic on people has still been persistent. Health has also become a vital problem concerned by countries and people all around the world. Under the background of normalization of international pandemic prevention and control, cultivating healthy lifestyle perceptions, implementing health-promoting lifestyle, and promoting higher life satisfaction need to be concerned and improved constantly.

Wuhan city in China, the outbreak place of the COVID-19 pandemic, is one of the most severely infected cities all over the world. On December 8, 2019, the COVID-19 pandemic was broke out in South China Seafood Market in Wuhan. With the rapid development of the pandemic, on January 23, 2020, Wuhan took the measure of “lockdown”. Until April 8, 2020, Wuhan city was “unsealed” and the COVID-19 pandemic was effectively under control. Amid the COVID-19 pandemic, people in Wuhan have deeply experienced and perceived the hazards and preventive measures of the COVID-19 pandemic. Health-promoting activities that are highly related to physical resistance and immunity have become the ardent demands of people (16). People are more eager to cultivate a healthy lifestyle (17). According to the survey of China Education Daily, 78% of people are willing to take exercise and maintain a healthy lifestyle after the COVID-19 pandemic (18). At the same time, Wuhan's medical and healthcare system has been gradually improved, mainly includes increasing financial investment and medical insurance, building a hierarchical medical system, enhancing the ability of grass-roots medical and health services, and improving the community joint prevention and control mechanism (19). Hence, it is convincing to select Wuhan residents as the survey sample to discuss the effect of the COVID-19 pandemic.

This study was built on the Health Belief Model (HBM), which was proposed by Hochbaum in 1958 (20) and improved by Becker and other social psychologists (21). HBM claimed that people's perceptions and behaviors of health would effectively maintain or promote people's health and further influence the achievement of people's self-satisfaction and self-actualization. That is, how people understand the severity and susceptibility of health and disease and how people take actions would directly influence people's self-satisfaction. Existing research studies have already shown that the COVID-19 pandemic has significantly aroused the people' health consciousness and effectively improved the rationality of people's health perceptions (22). Thus, this study is aimed to explore and compare the responses of people's health-promotion lifestyle and life satisfaction on the influence of the COVID-19 pandemic, that is, whether the people's health-promoting lifestyle and life satisfaction have been impacted by the COVID-19 pandemic. If so, what will happen? This study is expected to make a contribution to a deeper understanding of people's health-promoting lifestyle and life satisfaction in the context of an extraordinary pandemic and also give the theoretical and practical implications for developing and applying HBM. Based on the above discussion, the research hypotheses H1–H4 as follows were put forward.

H1: Residents' health-promoting lifestyle has been significantly influenced by the COVID-19 pandemic;

H2: Residents' life satisfaction has been significantly influenced by the COVID-19 pandemic;

H3: Residents' health-promoting lifestyle and life satisfaction have significant differences in demographic variables under the influence of the COVID-19 pandemic;

H4: Before and after the COVID-19 pandemic, residents' health-promoting lifestyle has a significantly impact on residents' life satisfaction.

## Methods

### Data Collection and Procedures

A questionnaire survey was the main survey method of this research. The residents who have resided in Wuhan for at least 1 year from 2019 and experienced the COVID-19 pandemic were selected as the respondents. The stratified convenient sampling method was mainly used to determine samples from 13 municipal districts of Wuhan (there are 13 municipal districts in Wuhan), namely, Hankou District, Hanyang District, Wuchang District, Dongxihu District, Caidian District, and so on. Then selected two communities in each municipal district and 70–100 residents in each community. Residents with cognitive impairment or serious diseases (such as mental illness and Alzheimer's disease) were excluded from the study. The questionnaire was distributed and collected from January to February in 2021 after the epidemic situation had been controlled and the residents had returned to normal life. For this study, the questionnaires were sent to the residents online by a professional survey app (so-jump). If participants have dyslexia, they would be interviewed offline by researchers. In the end, 2,054 valid samples were collected.

### Instrumentation

The questionnaire measured the following constructs: demographic survey, health-promoting lifestyle, and life satisfaction. The demographic survey was designed with six items to learn about residents' gender, age, education, marital status, permanent residence, and average family income.

#### Health-Promoting Lifestyle Profile

The Chinese version of Health-Promoting Lifestyle Profile (HPLP) by Huang (23) was adopted as the survey tool to measure residents' health-promoting lifestyle in this study, which was initially compiled by Walker et al. (4). The scale includes six dimensions and 42 items, namely, self-actualization (14 items), health responsibility (nine items), stress management (six items), interpersonal support (five items), nutrition (five items), and exercise (three items) (23). The survey utilized the four-point Likert scale from one (never) to four (routinely). A higher score of HPLP indicates a more excellent health-promoting lifestyle. The original Chinese version of HPLP by Huang has good reliability, in which the Cronbach's alpha of the scale was 0.930 and the Cronbach's alpha of the subscales was from 0.736 to 0.922 (23). In this research, the Cronbach's alpha was 0.922, which also shows good reliability.

#### Satisfaction With Life Scale

The Satisfaction with Life Scale (SWLS) as the survey tool to measure residents' life satisfaction was adopted in this study, which was initially compiled by Diener et al. (24). Five items were designed, i.e., “*In most ways, my life is close to my ideal*”, “*The conditions of my life are excellent*”, “*I am satisfied with my life*”, “*So far I have gotten the important things I want in life*”, and “*If I could live my life over, I would change almost nothing*” (24). The survey was utilized the seven-point Likert scale from one (strongly disagree) to seven (strongly agree). The original SWLS has a good reliability with the Cronbach's alpha of 0.870. In this research, the Cronbach's alpha was 0.813 with a good reliability.

### Data Analysis

EpiData was used to input and check the data. Data analysis was conducted by Stata 16.0. The respondents' demographic situation was statistically calculated by means, SD, and percentages. Paired groups were compared by paired sample *t*-test and one-way ANOVA. Person correlation analysis was used to measure the correlation between health-promoting lifestyle and life satisfaction. The significance level for all statistical analyses was set at *p* < 0.05 (two-tailed test). Multiple regression analyses were performed to explore the relative contribution of each significant variable. Health-promoting lifestyle and demographic variables were set as independent variables, and life satisfaction was set as dependent variables.

## Results

### General Characteristics

Of the total 2,200 anonymous questionnaires, 2,080 were returned and the response rate was 94.5%. In total, 26 invalid questionnaires were rejected due to the incomplete information, and 2,054 questionnaires were effective and the effective rate was 93.4%.

Table 1 shows the demographic characteristics of the survey samples. The proportion of men and women participating in the survey is balanced, with men for 49.1% and women for 50.9%. The proportion of each group's age is relatively balanced, and most of them have a high school diploma or higher, accounting for 79.3%. Unmarried and married groups are the main groups in the total sample. As far as permanent residence is concerned, the number of residents living in urban areas is slightly more than in the suburbs. The average family income is mostly beyond 40,000 RMB (see Table 1).

**Table 1:** General characteristics of participants (*N* = 2,054).

**Variable**	***N* (%)**	**M ±SD**
**Gender**
Men	1,009 (49.1%)	1.51 ± 0.50
Women	1,045 (50.1%)	
**Age**
18 and below	398 (19.4%)	3.06 ± 1.66
19–30	374 (18.2%)	
31–40	349 (17.0%)	
41–50	301 (14.7%)	
51–60	363 (17.7%)	
61 and above	269 (13.1%)	
**Education**
Elementary school or less	155 (7.5%)	3.27 ± 1.04
Middle school	291 (14.2%)	
High school	706 (34.4%)	
Post-secondary school and above	902 (43.9%)	
**Marital status**
Discoverture	1,070 (52.1%)	1.56 ± 0.66
Married	853 (41.5%)	
Divorced	100 (4.9%)	
Widowed	31 (1.5%)	
**Permanent residence**
Urban	1,110 (54.0%)	1.31 ± 0.46
Rural	944 (46.0%)	
**Average family income**
10,000 and below	376 (18.3%)	4.43 ± 2.22
10,001–20,000	221 (10.8%)	
20,001–30,000	109 (5.3%)	
30,001–40,000	95 (4.6%)	
40,001–50,000	392 (19.1%)	
50,001–60,000	403 (19.6%)	
60,001 and above	458 (22.3%)	

*N stands for the sample size; M stands for mean (the average value); SD stands for standard deviation. Average family income (RMB)*.

### Comparison of Health-Promoting Lifestyle and Life Satisfaction Before and After the COVID-19 Pandemic

Table 2 shows the changes in residents' health-promoting lifestyle and life satisfaction scores before and after the COVID-19 pandemic. The results show that health-promoting lifestyle [*t* = −3.67, *p* < 0.001] and life satisfaction [*t* = −2.57, *p* < 0.01] have changed significantly after the COVID-19 pandemic but the score of life satisfaction has shown a downward trend. The score of health-promoting lifestyle before the COVID-19 pandemic was [107.35 ± 24.08] and after was [108.16 ± 24.10] relatively. The score of life satisfaction before and after the COVID-19 pandemic was [21.12 ± 6.53] and [20.87 ± 6.60] relatively. Hypotheses 1 and 2 were verified.

**Table 2:** Comparison of Health-Promoting Lifestyle Profile (HPLP) and Satisfaction with Life Scale (SWLS) before and after the COVID-19 pandemic (*N* = 2,054).

**Variable**	**M** **±SD**		***t* and *P***
	**Before**	**After**	
HPLP	107.35 ± 24.08	108.16 ± 24.10	−3.67^***^
Health responsibility	17.62 ± 5.06	17.89 ± 4.98	−0.26
Exercise	12.73 ± 3.81	12.80 ± 3.73	−3.77^***^
Nutrition	18.13 ± 4.70	18.33 ± 4.73	−1.22
Self-actualization	19.09 ± 6.67	19.11 ± 4.70	−3.05^**^
Interpersonal support	21.44 ± 5.35	21.55 ± 5.41	−1.52
Stress management	18.34 ± 4.71	18.49 ± 4.70	−2.42^*^
SWLS	21.12 ± 6.53	20.87 ± 6.60	2.57^**^

* *p < 0.05*.

** *p < 0.01*.

*** *p < 0.001*.

*M stands for mean (the average value); SD stands for standard deviation*.

Among the six dimensions of health-promoting lifestyle, it can be seen that the scores of exercise [*t* = −3.77, *p* < 0.001], self-actualization [*t* = −3.05, *p* < 0.001], and stress management [*t* = −2.42, *p* < 0.05] have increased significantly after the COVID-19 pandemic. The score of exercise before the COVID-19 pandemic was [12.73 ± 3.81] and after the COVID-19 pandemic was [12.80 ± 3.73]. The score of self-actualization before the COVID-19 pandemic was [19.09 ± 6.67] and after the COVID-19 pandemic was [19.11 ± 4.70]. The score of stress management before the COVID-19 pandemic was [19.09 ± 6.67] and after the COVID-19 pandemic was [19.11 ± 4.70]. The scores of health responsibility, nutrition, and interpersonal support have increased but not significantly after the COVID-19 pandemic. The score of health responsibility before the COVID-19 pandemic was [17.62 ± 5.06] and after the COVID-19 pandemic was [17.89 ± 4.98]. The score of nutrition before the COVID-19 pandemic was [18.13 ± 4.70] and after the COVID-19 pandemic was [18.33 ± 4.73). The score of interpersonal support before the COVID-19 pandemic was [21.44 ± 5.35] and after the COVID-19 pandemic was [21.55 ± 5.41] (see Table 2).

### Comparison of HPLP and SWLS With General Characteristics Before and After the COVID-19 Pandemic

Table 3 shows the differences in residents' life satisfaction and health-promoting lifestyle in the different demographic variables before and after the COVID-19 pandemic.

**Table 3:** Comparison of HPLP and SWLS with general characteristics before and after the COVID-19 pandemic (*N* = 2,054).

**Variable**	**SWLS**		**t(F) and** ***p***		**HPLP**		**t(F) and** ***p***	
	**Before**	**After**	**Before**	**After**	**Before**	**After**	**Before**	**After**
**Gender**
Men	21.26 ± 6.87	21.02 ± 7.01	−1.99^*^	1.82^*^	107.17 ± 22.350	107.31 ± 22.46	−0.34	−1.58
Women	20.83 ± 5.70	20.53 ± 5.56			107.53 ± 25.643	108.99 ± 25.56		
**Age**
18 and below	23.08 ± 6.81	22.07 ± 7.11	11.27^***^	3.91^**^	108.96 ± 26.93	109.88 ± 26.42	3.26^**^	2.67^*^
19–30	21.50 ± 6.37	21.02 ± 6.50			107.07 ± 23.47	108.48 ± 23.96		
31–40	20.84 ± 6.01	20.95 ± 6.05			108.86 ± 22.80	109.03 ± 22.08		
41–50	20.44 ± 6.39	20.54 ± 6.37			105.54 ± 24.12	107.19 ± 23.65		
50–60	19.47 ± 7.64	19.92 ± 7.86			103.24 ± 17.56	103.54 ± 17.87		
61 and above	20.14 ± 5.28	20.17 ± 5.12			110.69 ± 28.11	109.91 ± 28.12		
**Education**
Elementary school or less	20.77 ± 6.43	20.89 ± 5.92	5.44^**^	3.39^**^	111.40 ± 27.44	110.81 ± 27.17	2.91^*^	2.58^*^
Middle school	20.29 ± 6.80	20.37 ± 6.73			104.15 ± 24.40	105.22 ± 23.88		
High school	20.61 ± 6.64	20.45 ± 6.88			106.82 ± 23.08	107.63 ± 22.75		
Undergraduate	21.56 ± 6.42	21.04 ± 6.51			107.64 ± 24.02	108.64 ± 24.20		
Master degree or above	22.67 ± 5.92	22.44 ± 6.04			109.82 ± 23.46	111.15 ± 24.44		
**Marital status**
Discoverture	21.86 ± 6.38	21.25 ± 6.48	10.21^***^	3.51^*^	107.43 ± 24.36	108.59 ± 24.58	0.08	0.96
Married	20.22 ± 6.59	20.32 ± 6.69			107.31 ± 23.06	108.02 ± 22.72		
Divorced	21.04 ± 6.83	21.53 ± 6.73			106.48 ± 23.06	104.42 ± 23.45		
Widowed	20.81 ± 6.51	20.65 ± 6.65			108.71 ± 40.92	109.52 ± 40.77		
**Permanent residence**
Urban	21.26 ± 6.88	21.02 ± 7.01	1.47	1.70	106.82 ± 22.64	107.76 ± 22.79	−1.40	−1.05
Rural	20.83 ± 5.70	20.53 ± 5.56			108.53 ± 26.93	109.04 ± 26.73		
**Average family income**
10,000 and below	22.15 ± 6.93	21.55 ± 7.13	9.77^***^	5.24^***^	104.92 ± 25.55	106.41 ± 25.29	3.01^**^	0.82
10,001–20,000	21.79 ± 5.91	21.87 ± 5.68			102.95 ± 21.42	106.28 ± 21.64		
20,001–30,000	23.40 ± 5.28	22.20 ± 5.75			106.48 ± 18.71	108.84 ± 19.76		
30,001–40,000		22.51 ± 6.60			106.54 ± 23.58	108.02 ± 22.81		
40,001–50,000		20.17 ± 6.10			108.45 ± 23.17	108.89 ± 22.95		
50,001–60,000		20.11 ± 6.61			108.78 ± 23.17	108.88 ± 23.28		
60,001 and above		20.43 ± 6.94			109.66 ± 26.39	109.13 ± 26.92		

* *p < 0.05*.

** *p < 0.01*.

*** *p < 0.001*.

*HPLP, Health-Promoting Lifestyle Profile; SWLS, Satisfaction with Life Scale; Average family income (RMB)*.

In terms of life satisfaction, before and after the COVID-19 pandemic, there were significant differences in the aspect of gender [*t*_before_ = −1.99, *p*_before_ < 0.05; *t*_after_ = 1.82, *p*_after_ < 0.05], age [*t*_before_ = 11.27, *p*_before_ < 0.001; *t*_after_ = 3.91, *p*_after_ < 0.01], education level [*t*_before_ = 5.44, *p*_before_ < 0.01; *t*_after_ = 3.39, *p*_after_ < 0.01], marital status [*t*_before_ = 10.21, *p*_before_ < 0.001; *t*_after_ = 3.51, *p*_after_ < 0.05], and average family income [*t*_before_ = 9.77, *p*_before_ < 0.001; *t*_after_ = 5.24, *p*_after_ < 0.001]. The individuals with the characteristics of men, younger age, higher education, unmarried, and middle average family income have significantly higher life satisfaction before and after the COVID-19 pandemic.

In terms of health-promoting lifestyle, before the COVID-19 pandemic, there were significant differences in terms of age [*t*_before_ = 3.26, *p*_before_ < 0.01], education level [*t*_before_ = 2.91, *p*_before_ < 0.05], and average family income [*t*_before_ = 3.01, *p*_before_ < 0.01]. After the COVID-19 pandemic, there were significant differences in health-promoting lifestyle scores in terms of age [*t*_after_ = 2.67, *p*_after_ < 0.05] and education level [*t*_after_ = 2.58, *p*_after_ < 0.05], but the average family income became less significant (see Table 3). Hypothesis 3 was partially verified.

### Correlations Between Health-Promoting Lifestyle and Life Satisfaction

Table 4 shows the correlation between health-promoting lifestyle and life satisfaction before and after the COVID-19 pandemic. The result shows that a health-promoting lifestyle is positively correlated with life satisfaction before and after the COVID-19 pandemic. The correlation coefficient between health-promoting lifestyle and life satisfaction is 0.33. All the six dimensions of a health-promoting lifestyle are positively correlated with life satisfaction before and after the COVID-19 pandemic. Before the COVID-19 pandemic, the correlation coefficients between life satisfaction and the six dimensions of self-actualization, health responsibility, exercise, nutrition, interpersonal support, and stress management are 0.37, 0.23, 0.21, 0.26, 0.31, and 0.28, respectively. After the COVID-19 pandemic, the correlation coefficients between life satisfaction and the six dimensions of self-actualization, health responsibility, exercise, nutrition, interpersonal support, and stress management are 0.35, 0.24, 0.22, 0.26, 0.30, and 0.30, respectively (see Table 4).

**Table 4:** Correlations between health-promoting lifestyle and life satisfaction (*N* = 2,054).

	**Variables**	**1**	**2**	**3**	**4**	**5**	**6**	**7**	**8**
Before	1.SWLS	1							
	2.Self-actualization	0.37^**^	1						
	3.Health responsibility	0.23^**^	0.62^**^	1					
	4.Exercise	0.21^**^	0.60^**^	0.71^**^	1				
	5.Nutrition	0.26^**^	0.62^**^	0.70^**^	0.70^**^	1			
	6.Interpersonal support	0.31^**^	0.71^**^	0.64^**^	0.62^**^	0.66^**^	1		
	7.Stress management	0.28^**^	0.67^**^	0.67^**^	0.65^**^	0.70^**^	0.75^**^	1	
	8.HPLP	0.33^**^	0.83^**^	0.85^**^	0.82^**^	0.86^**^	0.87^**^	0.87^**^	1
After	1.SWLS	1							
	2.Self-actualization	0.35^**^	1						
	3.Health responsibility	0.24^**^	0.61^**^	1					
	4.Exercise	0.22^**^	0.60^**^	0.69^**^	1				
	5.Nutrition	0.26^**^	0.64^**^	0.67^**^	0.70^**^	1			
	6.Interpersonal support	0.30^**^	0.71^**^	0.65^**^	0.65^**^	0.69^**^	1		
	7.Stress management	0.30^**^	0.66^**^	0.66^**^	0.67^**^	0.71^**^	0.74^**^	1	
	8.HPLP	0.33^**^	0.82^**^	0.84^**^	0.83^**^	0.86^**^	0.88^**^	0.87^**^	1

* *p < 0.05*.

** *p < 0.01*.

*** *p < 0.001*.

*HPLP, Health-Promoting Lifestyle Profile; SWLS, Satisfaction with Life Scale*.

### Analysis of Influencing Factors of Life Satisfaction Before and After the COVID-19 Pandemic

Table 5 shows the results of multiple regression analysis on life satisfaction before and after the COVID-19 pandemic. The statistically significant demographic variables and the scores of each dimension of health-promoting lifestyle were used as independent variables, and the total score of life satisfaction was used as the dependent variable to perform multiple linear regression analysis. The results show that the health-promoting lifestyle, age, marital status, average family income, permanent residence, and self-actualization have a significant impact on life satisfaction before the COVID-19 pandemic and the explanation for the total variation in life satisfaction is 16.1% (overall model adjust *R*^2^ = 0.16, *p* < 0.001). After the COVID-19 pandemic, the health-promoting lifestyle, age, marital status, average family income, permanent residence, self-actualization, and stress management have a significant impact on life satisfaction. The explanation for the total variation in life satisfaction is 14% (overall model adjust *R*^2^ = 0.14, *p* < 0.001; see Table 5). Hypothesis 4 was verified.

**Table 5:** Analysis of influencing factors of life satisfaction before and after the COVID-19 pandemic.

		**Unstandardized**		**Standardized**	
	**Independent variables**	**B**	**SE**	**β**	**t(F) and *p***
Before	HPLP	0.09	0.01	0.33	15.63^***^
	Age	−0.51	0.13	−0.13	−4.03^***^
	Marital status	0.65	0.30	0.07	2.17^*^
	Average family income	−0.31	0.07	−0.11	−4.57^***^
	Permanent residence	−0.61	0.29	−0.04	−2.11^*^
	Self-actualization	0.41	0.04	0.30	9.42^***^
After	HPLP	0.10	0.02	0.33	15.63^***^
	Age	−0.31	0.13	−0.08	−2.38^***^
	Marital status	0.86	0.31	0.09	2.80^***^
	Average family income	−0.28	0.07	−0.09	−4.10^***^
	Permanent residence	−0.66	0.30	0.05	−2.21^*^
	Self-actualization	0.36	0.04	0.25	8.08^***^
	Stress management	0.15	0.05	0.11	3.04^***^

*Over Model R^2^ = 0.166; adjust R^2^ = 0.16 (before the COVID-19 pandemic)*.

*Over Model R^2^ = 0.145; adjust R^2^ = 0.14 (after the COVID-19 pandemic)*.

* *p < 0.05*.

** *p < 0.01*.

*** *p < 0.001*.

*Independent variables, health-promoting lifestyle; Dependent variables, life satisfaction; HPLP, Health-Promoting Lifestyle Profile*.

## Discussion

This study has investigated the relationship between health-promoting lifestyle and life satisfaction before and after the COVID-19 pandemic. The result has shown that after the outbreak of the COVID-19 pandemic, the residents' total score and the scores of all dimensions of health-promoting lifestyle are higher than those before the pandemic. This is consistent with the existing findings (25). That is, after the outbreak of the COVID-19 pandemic, people are paying more attention to the multidimensional pattern of self-initiated actions and perceptions, such as health responsibility, exercise, nutrition, self-actualization, interpersonal support, and stress management. The government and non-governmental organizations have formulated corresponding plans and issued relevant policies to increase the opportunities for residents to participate in physical exercise and develop a healthy lifestyle. The government of Wuhan has issued the *Home scientific fitness guide*, which updates the new standards of residents' physical practice and increases the types of indoor physical activities (such as yoga and aerobics) to provide scientific guidance (26). In addition to the government, some technology companies have launched the “Internet + sports” model and fitness apps, such as “Keep”. These fitness apps have successively set up the modules, such as “Online marathon” or “Online training camp”, to encourage residents to participate indoors (27). Communities have actively organized activities to encourage residents to participate in physical exercise, such as community marathons, fun sports meetings, and health promotion meetings (28). According to the HBM, this may be closely related to the change of positive attitude toward health (29). The consciousness and attitude of how people treat health and disease would directly influence people's healthy actions, further influencing self-satisfaction. This is also consistent with the viewpoint of emphasizing the importance of psycho-physic-mental continuity for overall health (30). After deep experiencing and perceiving the COVID-19 pandemic, residents' consciousness and perceptions of disease and health would be significantly improved and further promote the health-promoting lifestyle.

The result has shown that residents' life satisfaction score after the COVID-19 pandemic was lower than that before the pandemic. This is consistent with the existing research (31). This could be caused by the diffusion of the COVID-19 pandemic, which not only produces an effect on the change of anxiousness of physical health, but also increases the psychological pressure. To some extent, pressure and anxiety will reduce life satisfaction (32).

Besides, based on the HBM, people's demographic characteristics would directly influence people's health-promoting lifestyle and life satisfaction. The results of this study have also shown that age, marital status, and average family income have a significant impact on residents' life satisfaction. Age and education level have a significant effect on residents' health-promoting lifestyle. This keeps pace with the previous studies (33).

In addition, the results of multiple linear regression show that the health-promoting lifestyle is an influencing factor on life satisfaction whether before or after the COVID-19 pandemic. Especially, self-actualization and stress management have a significant positive influence on life satisfaction. This study has identified self-actualization as an important influencing factor on life satisfaction, which is inconsistent with the research results of Sak et al. (13). The reason may be that individuals with higher self-actualization have stronger psychological endurance, which is not easily affected by anxiety and fear. For the panic caused by COVID-19, these groups will usually maintain an optimistic attitude, be good at adjusting their emotions, and give positive psychological hints for themselves. Stress management has been proved as a vital influencing factor on residents' life satisfaction, which is consistent with Yang's research results (34). Studies have shown that improving one's ability to withstand stress could deal with more external challenges and enhance self-efficacy to face difficulties actively (35).

## Conclusion

This study mainly explores and analyzes the changes in health-promoting lifestyle and life satisfaction and the essential predictors that affect life satisfaction before and after the COVID-19 pandemic. It also demonstrates the relationship between health-promoting lifestyle and life satisfaction. These findings have suggested that the health-promoting lifestyle has been impacted significantly by the COVID-19 pandemic. In addition, the health-promoting lifestyle has improved after the pandemic, but the score of life satisfaction has shown a downward trend. Before and after the COVID-19 pandemic, the health-promoting lifestyle has a significant impact on residents' life satisfaction.

In response to the impact of the COVID-19 pandemic, on September 16, 2020, the United Nations has released *The United Nations Comprehensive Response to the COVID-19 Report* (36). It has advocated that governments and health organizations of all countries should play a leading role in global health countermeasures, fully mobilize every family to cultivate health awareness, help people form a healthy lifestyle, and cultivate more healthy behaviors. Therefore, a variety of measures should be taken to promote a health-promoting lifestyle in the post-pandemic period. Such as promoting health publicity through various channels; strengthening health education in terms of self-actualization, health responsibility, exercise, nutrition, interpersonal support, and stress management to establish the correct concept of health; promoting the quality of health-related social services, and carrying out more health-related activities to increase residents' enthusiasm and chances to improve healthy lifestyle; strengthening psychological guidance and improving psychological endurance to enhance the stress management ability and help residents improve their life satisfaction.

This study has the following limitations. The survey sample is restricted to the city of Wuhan. Although the city of Wuhan has the typical representativeness, it does not have general applicability. Follow-up research can further expand the scope of the investigation and select regions with different pandemic levels for comparative analysis. In addition, a deeper investigation of residents' perceptions and behaviors will be conducted in a follow-up study.

## Data Availability Statement

The raw data supporting the conclusions of this article will be made available by the authors, without undue reservation.

## Ethics Statement

The questionnaire data were collected anonymously in this study, and questionnaire filling was voluntary. The subjects were promised that the questionnaire data would only be used for academic research and kept strictly confidential. The participants have provided their written informed consent to participate in this study.

## Author Contributions

DK: data analysis and reporting and manuscripts. WC: reviewing and editing and supervision. Both authors contributed to the article and approved the submitted version.

## Conflict of Interest

The authors declare that the research was conducted in the absence of any commercial or financial relationships that could be construed as a potential conflict of interest.

## Publisher's Note

All claims expressed in this article are solely those of the authors and do not necessarily represent those of their affiliated organizations, or those of the publisher, the editors and the reviewers. Any product that may be evaluated in this article, or claim that may be made by its manufacturer, is not guaranteed or endorsed by the publisher.
